# Supplementary figures and images for: Cryo-EM structures of the human PA200 and PA200-20S complex reveal regulation of proteasome gate opening and two PA200 apertures
Source: PLoS Biol. 2020 Mar 5;18(3):e3000654. doi: 10.1371/journal.pbio.3000654 (PMC7077846; doi:10.1371/journal.pbio.3000654)

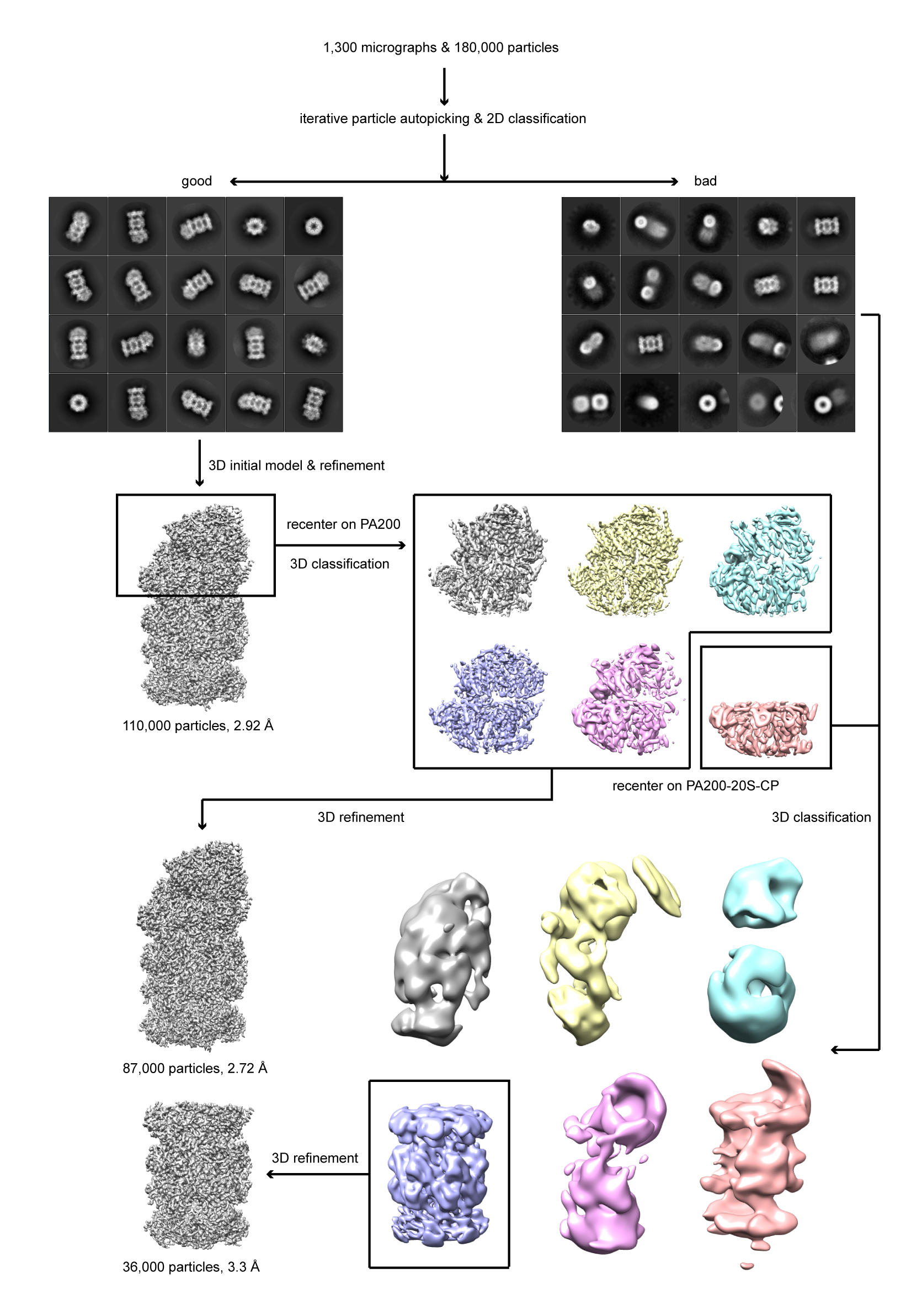

Supplement: S1 Fig — We applied routine processing including classification and refinement to obtain particles’ orientation, then reextracted subparticles consisting of whole PA200 and low density of 20S-CP interacting with PA200 by calculating a vector in pixels from the whole complex to the center of PA200. The subparticles were subjected to another 3D classification without alignment, and only particles with good features of PA200 were selected for the final reconstruction, CTF refinement, and postprocessing. CP, core particle; CTF, contrast transfer function; PA200, proteasome activator 200. (TIF) [file pbio.3000654.s001.tif]

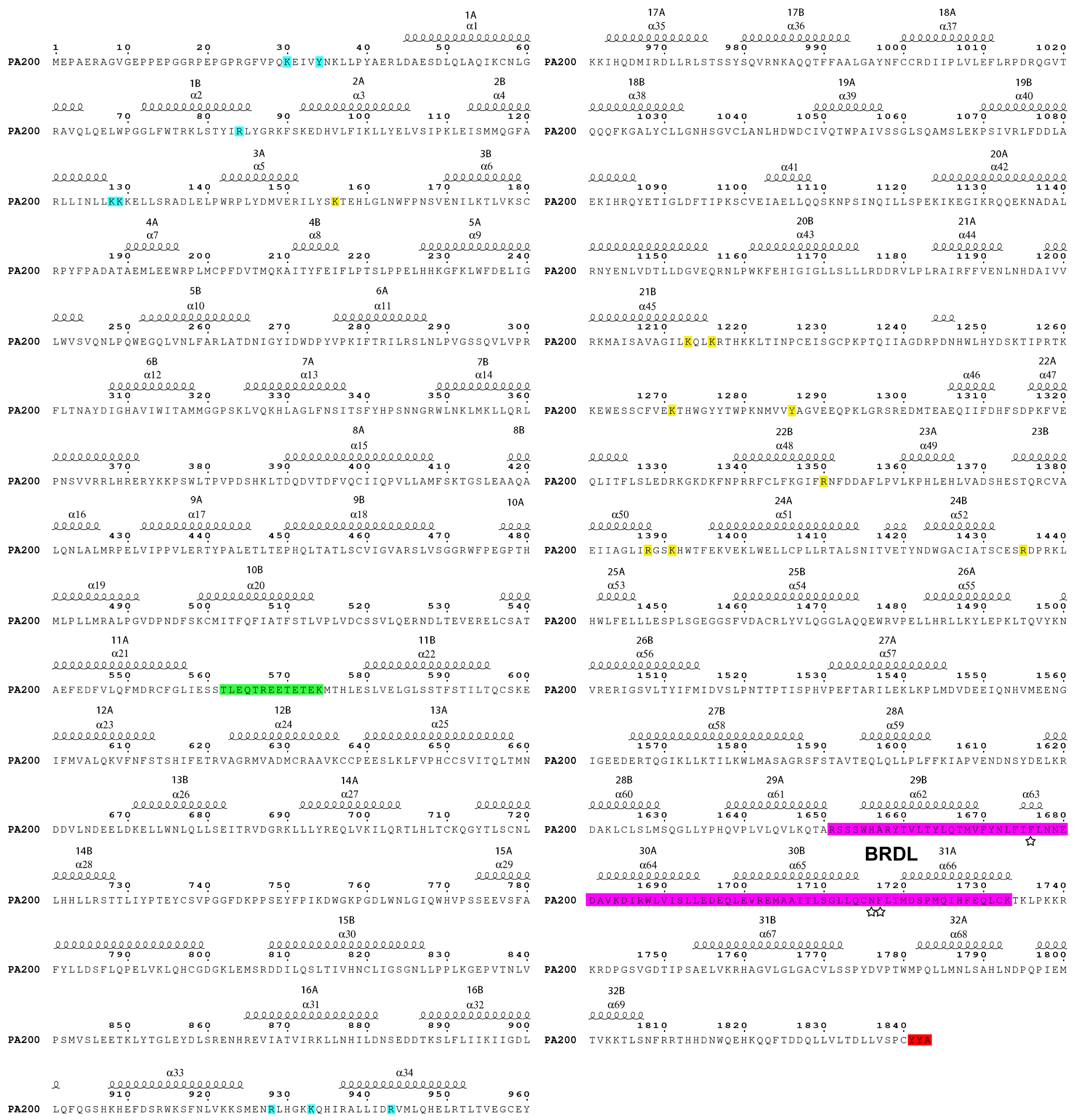

Supplement: S2 Fig — HEAT repeat helices are labeled 1A for helix A of HEAT repeat 1, etc. The BRDL domain of PA200 is colored magenta. The C-terminal (YYA) and the loop (Thr562 to Lys574) inserted into the 20S are colored red and green, respectively. The residues involved in the interaction between PA200 and 5,6[PP]-InsP4 and InsP6 are colored cyan and yellow. The key residues of BRDL are indicated by ☆. 5,6[PP]2-InsP4, (5,6)-bisdiphosphoinositol tetrakisphosphate; BRDL, bromodomain-like; InsP6, inositol hexakisphosphate; PA200, proteasome activator 200. (TIF) [file pbio.3000654.s002.tif]

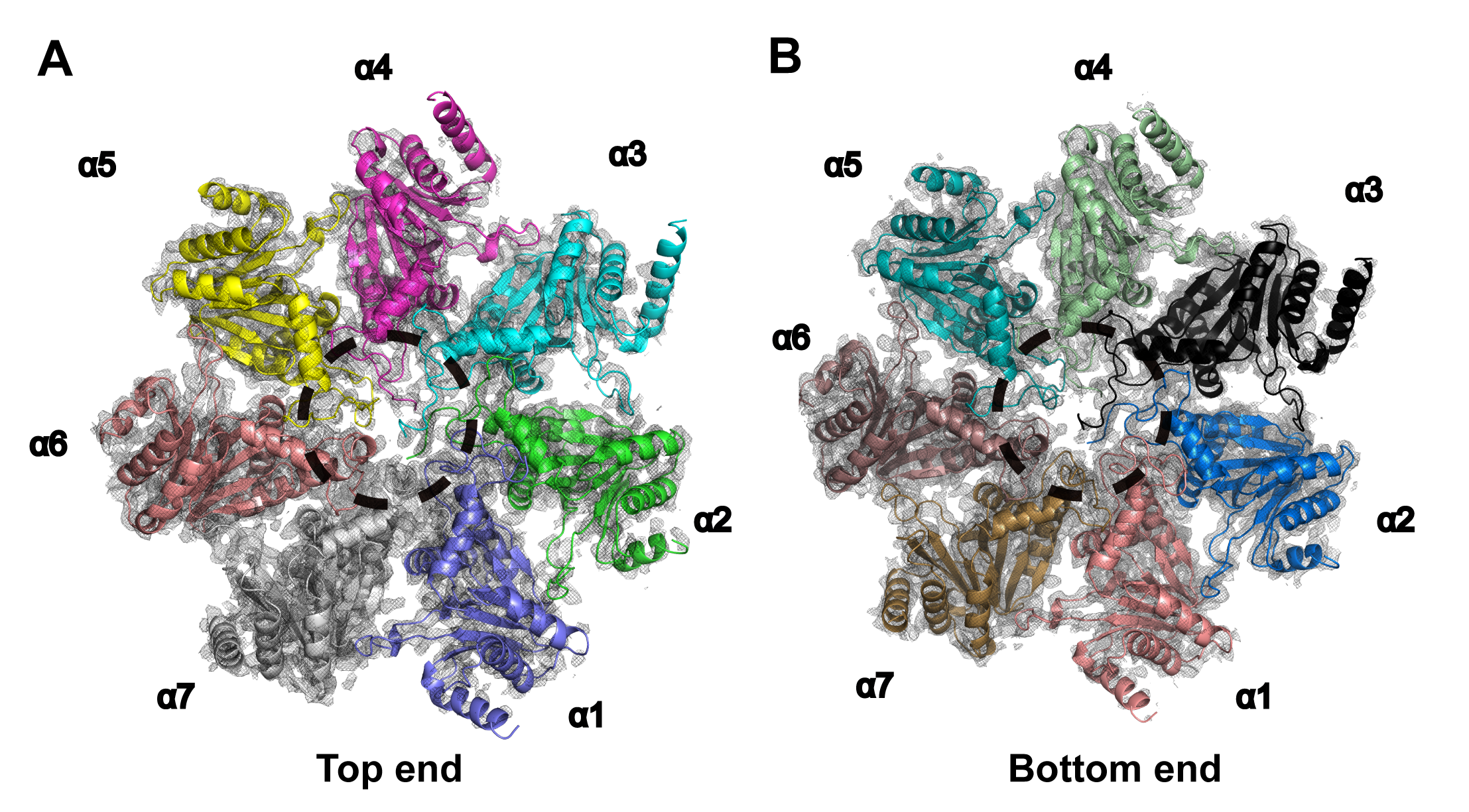

Supplement: S3 Fig — (A-B) Close-up views of the two α-rings (A: top end; B: bottom end). The cryo-EM maps are shown as gray mesh and the atomic models as cartoon. Both gates of the two α-rings are closed and indicated with a dotted circle. cryo-EM, cryo–electron microscopy; PA200, proteasome activator 200. (TIF) [file pbio.3000654.s003.tif]

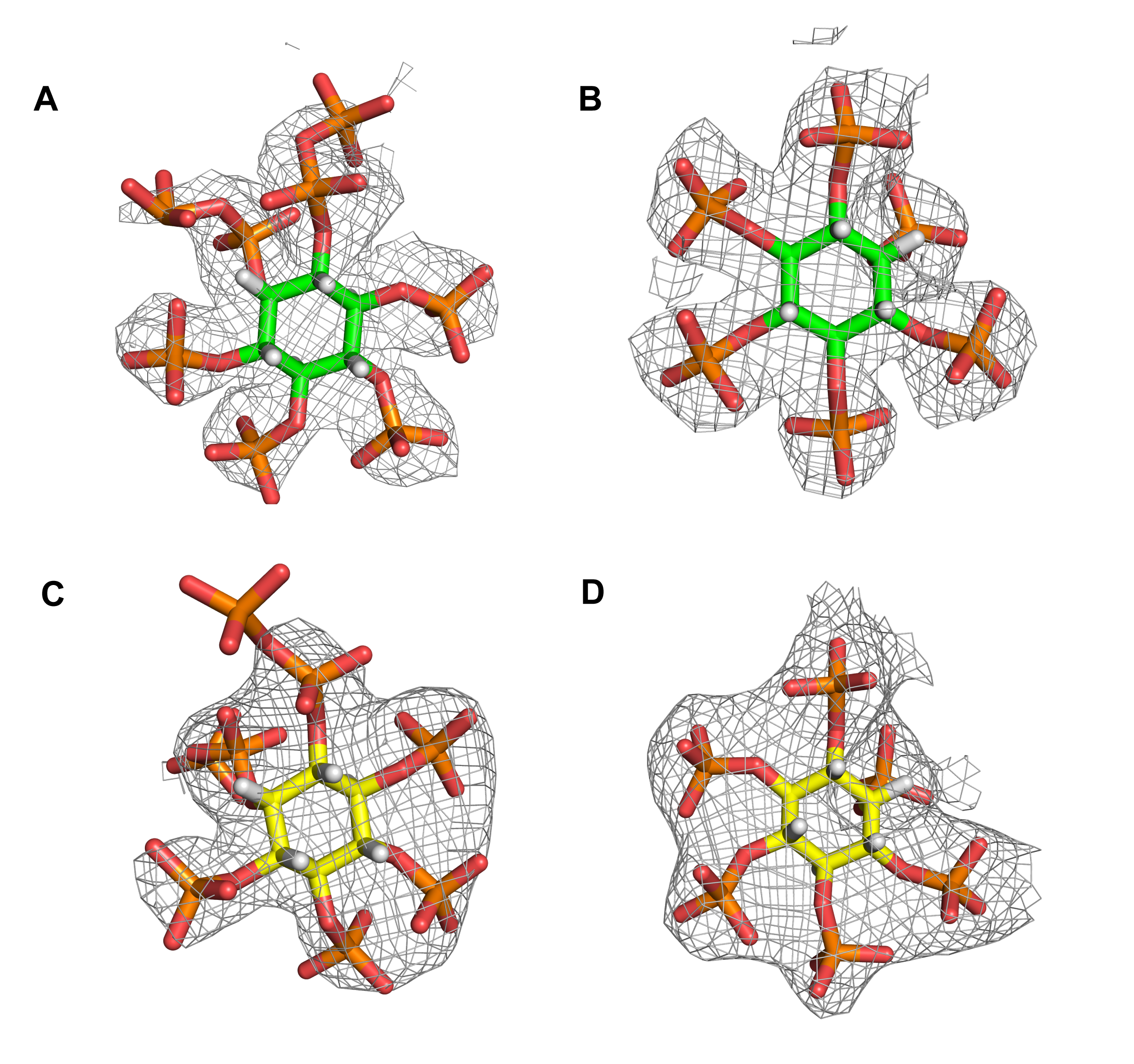

Supplement: S4 Fig — (A-B) Cryo-EM densities bound by the openings of PA200 in the complex are fitted with 5,6[PP]-InsP4 (A, opening 1) and InsP6 (B, opening 2), respectively. (C-D) Cryo-EM densities bound by the openings of apo PA200 are fitted with 5,6[PP]-InsP4 (A, opening 1) and InsP6 (B, opening 2), respectively. 5,6[PP]2-InsP4, (5,6)-bisdiphosphoinositol tetrakisphosphate; cryo-EM, cryo–electron microscopy; InsP6, inositol hexakisphosphate; PA200, proteasome activator 200. (TIF) [file pbio.3000654.s004.tif]

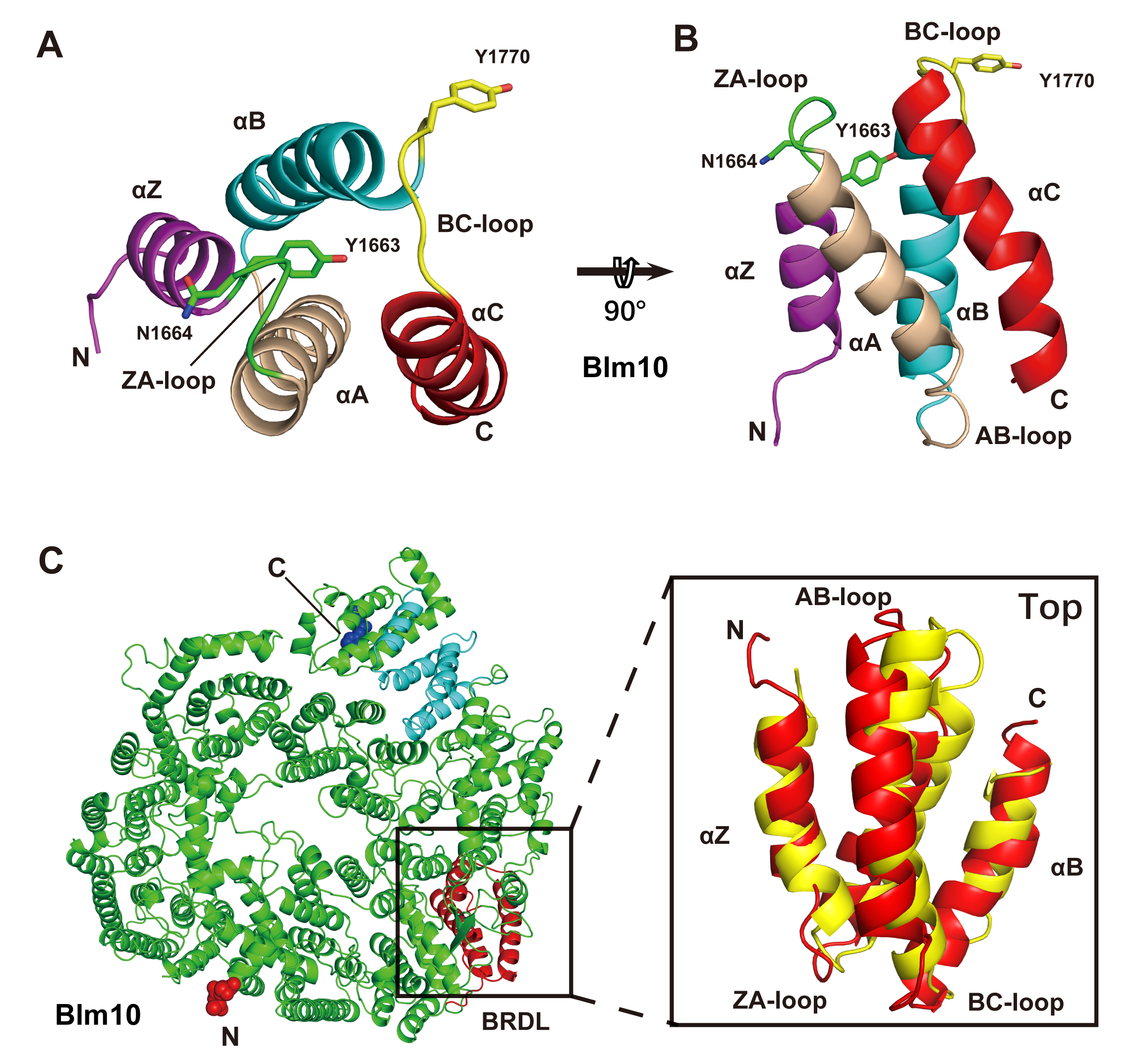

Supplement: S5 Fig — (A-B) Same as Fig 6A and 6B, panel A shows the bottom view Blm10 BRDL domain (PDB: 4V7O), whereas panel B shows the side view. (C) Same as Fig 6C, the BRDL domain of Blm10 is shown in red and superpositioned with that of its PA200 counterpart (yellow) in the right black box. The N-terminal of Blm10 is shown as red spheres, whereas the C-terminal is indicated as blue spheres. Blm10, Bleomycin resistance 10; BRDL, bromodomain-like; PA200, proteasome activator 200; PDB, Protein Data Bank. (TIF) [file pbio.3000654.s005.tif]

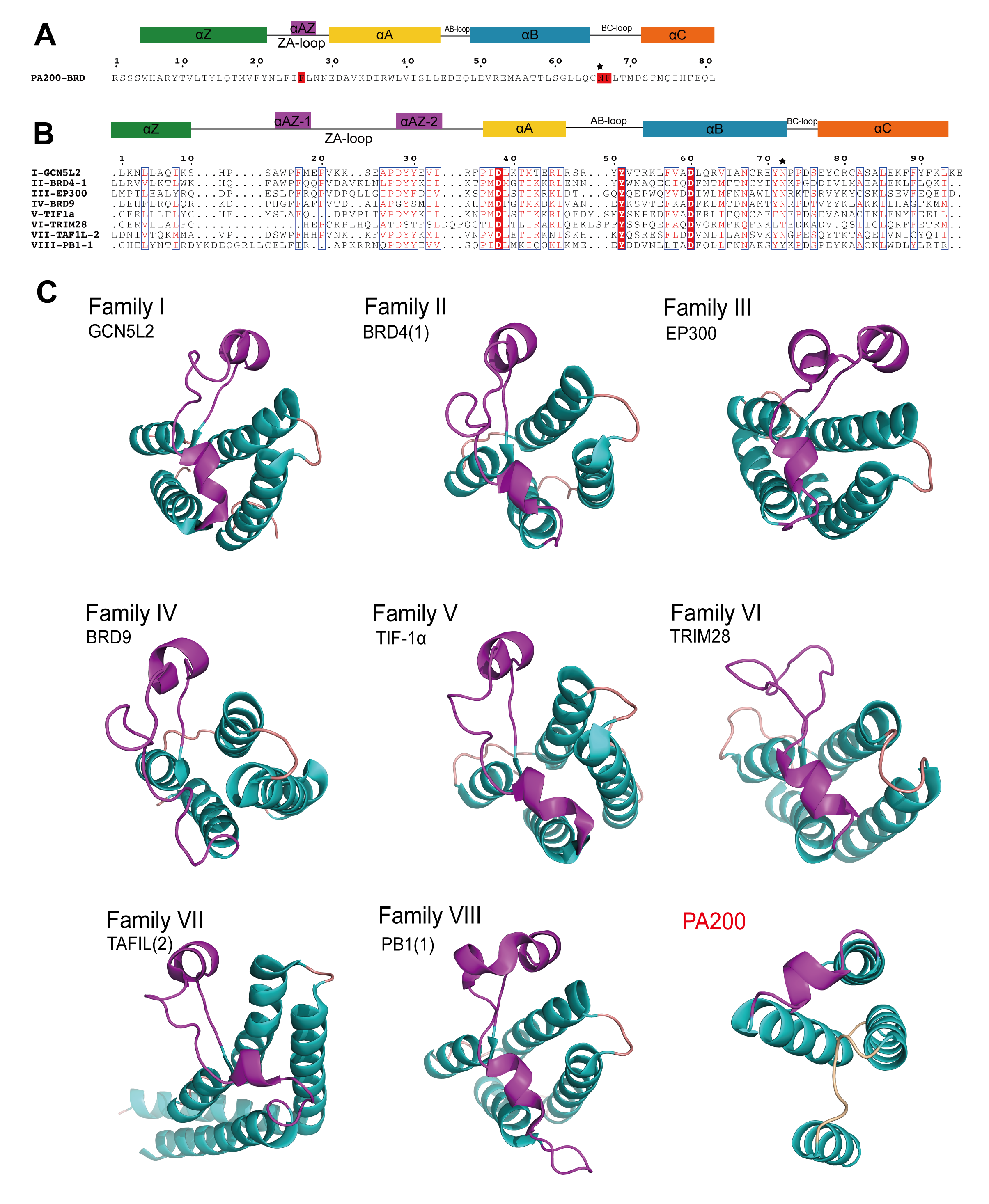

Supplement: S6 Fig — (A) Schematic drawing of PA200 BRDL domain. Key residues are colored red, whereas a black star indicates the Asn residue that binds acetyl-Lys. (B) Domain organization and the sequence alignment of the typical human BRD families. (C) Ribbon diagrams of eight BRD families and PA200. PDB code: GCN5L2 (3D7C), BRD4(1) (2OSS), EP300 (3I3J), BRD9 (3HME), TIF1α (2YYN), TRIM28 (2RO1), TAF1L(2) (3HMH), PB1(1) (3IU5). BRD, bromodomain; BRDL, BRD-like; PA200, proteasome activator 200; PDB, Protein Data Bank. (TIF) [file pbio.3000654.s006.tif]

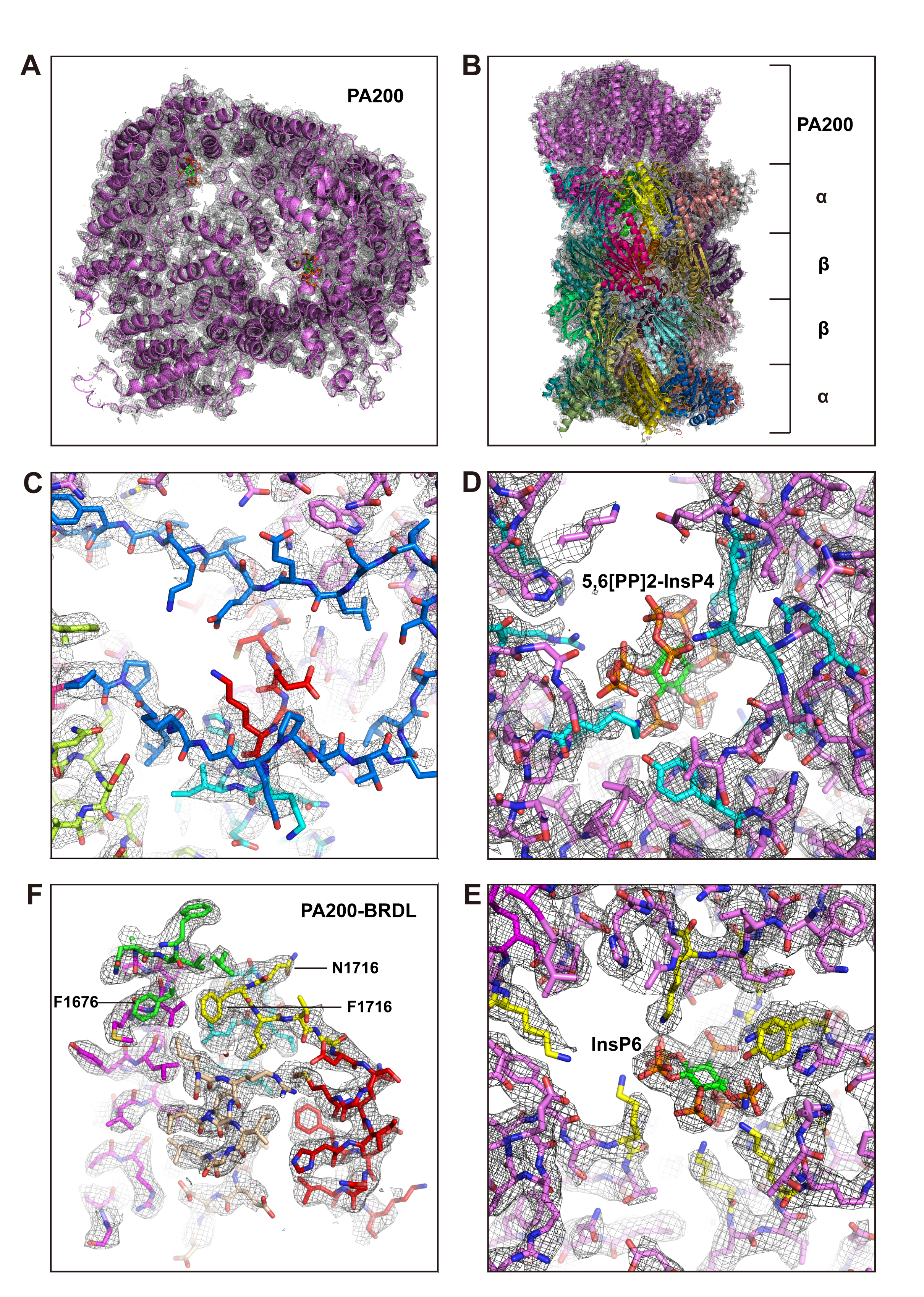

Supplement: S7 Fig — (A) Cryo-EM map (gray mesh) of the recombinant human PA200 with a fitted atomic model. (B) Cryo-EM map (gray mesh) of the recombinant human PA200-20S complex with a fitted atomic model. (C) A rectangular lid covering the region of PA200 previously reported as a big lateral opening (13 × 22 Å) located at the dome-like structure of Blm10. Close-up views of the cryo-EM map (gray mesh) with a fitted atomic model (cartoon representation) of the lid. (D-E) Close-up views of the cryo-EM map (gray mesh) with a fitted atomic model (cartoon representation) for the two openings and inositol phosphate cofactors (D: opening 1, E: opening 2). (F) Close-up views of the cryo-EM map (gray mesh) with a fitted atomic model (cartoon representation) for the BRDL domain. Blm10, Bleomycin resistance 10; BRD, bromodomain; BRDL, BRD-like; cryo-EM, cryo–electron microscopy; PA200, proteasome activator 200. (TIF) [file pbio.3000654.s007.tif]
