# Supplementary material for: Cryo-EM structures of the human PA200 and PA200-20S complex reveal regulation of proteasome gate opening and two PA200 apertures
Source: PLoS Biol. 2020 Mar 5;18(3):e3000654. doi: 10.1371/journal.pbio.3000654 (PMC7077846; doi:10.1371/journal.pbio.3000654)
Supplement: S1 Table — (DOCX) [file pbio.3000654.s008.docx]

**Table S1. Data collection and refinement statistics**

| **Dataset** | **PA200** | **PA200-20S** |
| --- | --- | --- |
| **Data collection** |  |  |
| EM equipment | FEI Titan Krios | FEI Titan Krios |
| Voltage (kV) | 300 | 300 |
| Detector | K2 Summit | K2 Summit |
| Pixel size (Å) | 1.04 | 1.04 |
| Electron dose (e^–^/Å^2^) | 60 | 60 |
| Defocus range (μm) | -1.8~-2.5 | -1.8~-2.5 |
| **Reconstruction** |  |  |
| Software | RELION 3.0 + cisTEM | RELION 3.0 + cisTEM |
| Particle Numbers | 103,000 | 87,000 |
| Symmetry | C1 | C1 |
| FSC threshold | 0.143 | 0.143 |
| Final resolution (Å) | 3.75 | 2.72 |
| Map-sharpening *B* factor (Å^2^) | -199.39 | -52.52 |
| **Model building** |  |  |
| Software | Coot | Coot |
| **Refinement** |  |  |
| Software | Phenix | Phenix |
| CC_mask | 0.7747 | 0.8037 |
| Bond lengths (Å) | 0.008 | 0.011 |
| Bond angles (°) | 1.488 | 1.193 |
| **Validation** |  |  |
| Clashscore | 7.69 | 5.58 |
| Rotamers outliers (%) | 2.35 | 0.95 |
| **Ramachandran plot** |  |  |
| Favored (%) | 85.66 | 93.97 |
| Allowed (%) | 13.41 | 5.85 |
| Outliers (%) | 0.93 | 0.18 |
