# Supplementary material for: Cryo-EM structures of the human PA200 and PA200-20S complex reveal regulation of proteasome gate opening and two PA200 apertures
Source: PLoS Biol. 2020 Mar 5;18(3):e3000654. doi: 10.1371/journal.pbio.3000654 (PMC7077846; doi:10.1371/journal.pbio.3000654)
Supplement: Attachment — Submitted filename: Response to reviewers.docx [file pbio.3000654.s012.docx]

**Point-to-point responses to reviewer’s comments**

**Rev. 1:**

Q1: The authors claim that PA200 is unable to bind both ends of the 20S proteasome even in high molar excesses in Figure1. However, they clearly find double-capped particles in negative stained images, which appear to be lost in the cryo preparation of Figure 1C. In my opinion the authors need to demonstrate if the second PA200 is lost as a consequence of grid preparation. They should show by if double capped particles exist and what the proportion of them is in comparison to the EM preparations.

***Response****:* We thank the reviewer for pointing out this issue. We apologize for the ambiguity of the text between lines 154-156 of the manuscript. We are not claiming that the PA200 is unable to bind to both ends of 20S proteasome as commented by the reviewer, but that the obtained sample exhibited significant heterogeneity, i.e. consisted of both single-capped PA200-CP and double-capped PA200_2_-CP.

In order to improve the clarity, we [rewrote](https://cn.bing.com/dict/search?q=rewrote&FORM=BDVSP6&mkt=zh-cn) the sentence (lines 155-158 of the revision) which now reads "Thus, the data displayed significant heterogeneity of PA200 binding with the 20S regarding the assembly of single- and double-capped complexes, even when the concentration of PA200 was more than four times higher than that of 20S in our experiment."

We also followed the reviewer’s suggestion to check the methods used in our experiment and the particles in our EM images. In our study, the standard holey grids were used for cryogenic sample preparation, and the double-capped complexes might have been enriched on the supporting carbon film. The double-capped particles can actually be observed in some EM images (please see the figures below), but the number of double-capped complex particles picked out from those images was insufficient for us to carry out the 3D reconstruction and to calculate their proportion. In addition, another recently published study of the PA200-20S proteasome made use of EM grids empirically coated with a thin layer of continuous carbon film, which contributes to lower image contrast but favors better distribution of particles that resulted in more readily observable double-capped complex particles [1]. Therefore, as the reviewer mentioned, the difference between EM grids may be one of the reasons why the double-capped particles appear to be underrepresented in the EM images in our study.

Q2: With regards to gate opening, they should determine a structure of double-capped PA200-20S to reveal if gate opening is indeed a consequence of PA200 binding or just an inherent assymmetry of the 20S proteasome. Related to this aspect, what effects do mutations of the YYA motif elicit in degradation assays?

***Response****:* Numerous 20S CP from plasmodium, yeast, human, and other mammals have been solved by X-ray or electron microscopy, and their overall structures and subunit arrangements are virtually identical. The two substrate entry channels of the dyad-symmetric 20S CP into the internal proteolytic chamber are usually locked by the N-terminal tails of specific α subunits (α2, α3 and α4) and form an ordered gate that must be opened for substrate entry [2-8]. The conserved HbYX motif located at the C-terminus of many proteasomal activators (e.g. the RP) and proteins are known to bind the α-ring surface of proteasome inserts into the pockets between neighboring α-subunits to trigger gate opening. In line with this, our structural data shows that the C-terminal (YYA) of PA200 plays a crucial role in the interaction between PA200 and 20S by using side chains of Tyr1841 and Tyr1842 to form hydrogen bonds with α5 Glu25 and α5 Gly19, which stabilizes the adjacent α5 Pro17 reverse turn and displaces it to activate proteasome gate opening. Furthermore, the cryo-EM structures of 20S complexed with four 7-8 aa peptides mimicking the mutated C-terminal HbYX motif of PAN have shown that these mutants fail to induce gate opening and thus are unable to facilitate substrate entry and degradation [9]. We propose that the mutations of the C-terminal YYA motif of PA200 will similarly impair the degradation of substrate by the PA200-20S proteasome.

To further confirm that the gate opening is a consequence of PA200 binding, we processed our data anew and obtained the density map of the PA200-free 20S at a final resolution of 3.3 Å. The paragraph describing this process was added under "Image processing" subsection of the methods, and reads as follows: "The sub-particles without density corresponding to PA200 were re-extracted from original images by calculating a vector from the center of PA200 to the center of the whole complex, combined with particles classified as 20S and bad particles in previews 2D class average procedure, and then subjected to 3D classification and refinement. This process produced the final 20S map with an overall resolution of 3.3 Å. "

The density map of the PA200-free 20S clearly shows that the gates on either end of the 20S are closed. In contrast to our map of the PA200-20S complex (**Fig 3B and 3D**), the gates were completely closed by the N-terminal tails of α2, α3 and α4 subunits (**please see the new** **Fig S3**). These results are consistent with the previous and recent studies [8].

On the basis of all the abovementioned data from our study and other relevant studies, we conclude that the gate opening in our complex is indeed a consequence of PA200 binding.

Q3: What is the function of the different inositol species on peptide degradation. Are the inositol phosphates required for the stimulation of proteolytic activity? Does it has to be a mixture of both inositol phosphates, or is one or the other sufficient? A further question that arises: Could the density for the 5,6(PP)2-InsP4 also be interpreted as InsP6 and two solvent molecules?

***Response****:* We appreciate the reviewer’s comment on this interesting and important point. We carefully calculated the densities of these two small molecules once more and are convinced that the densities represent two different molecules and correspond to 5,6[PP]2-InsP4 (C_6_H_20_O_30_P_8_) and InsP6 (C_6_H_18_O_24_P_6_). To validate our structural data, standard InsP6 (phytic acid sodium salt hydrate, C_6_H_18_O_24_P_6_ · xNa^+^ · yH_2_O, MERCK, P8810) and purified PA200 (20 mM HEPES, pH 7.5 and 150 mM NaCl, 1mM DTT) were boiled for 5 minutes at 95°C and analyzed by HPLC-MS. The standard InsP6 produced a characteristic peak at 814.85 (M_InsP6_+7Na)^+^ (**please see the new Fig 6A**), and PA200 produced several characteristic peaks. In addition to the characteristic peak of 814.85 (M_InsP6_+7Na)^+^ in PA200, several other peaks representing different forms of sodium 5,6[PP]2-InsP4 with varying number of Na^+^ were observed, such as peak 1019.19 (M_5,6[PP]2-InsP4_+9Na)^+^, peak 1041.13 (M_5,6[PP]2-InsP4_+10Na)^+^ and peak 1063.24 (M_5,6[PP]2-InsP4_+11Na)^+^ (**please see the new Fig 6B**).

The paragraph describing this result was added under "**Two openings on PA200 bind inositol phosphate cofactors**" subsection of the results.

Previous studies have shown that Ins(1,4,5,6)P4 is a bona fide conserved regulator of class I histone deacetylases (HDACs), which acts as an ‘intermolecular glue’ between HDAC3 and its co-repressor SMRT [10-12]. The inositol phosphates can regulate the enzymatic activity of HDACs by binding within a pocket sandwiched between the HDAC and co-repressor proteins. In order to evaluate whether the inositol phosphates are required for the stimulation of proteolytic activity, we attempted to remove InsP6 and 5,6[PP]2-InsP4 from PA200 by treating the protein with a high-salt buffer. Unfortunately, we failed to obtain the inositol phosphate-free PA200 even when using a buffer containing 1 M NaCl. This result is consistent with our structure, which shows that all phosphate groups of InsP6 and 5,6[PP]2-InsP4 interact with positively charged residues of PA200, suggesting a robust binding affinity between these two molecules and PA200. The exact function of the two molecules thus requires further investigation.

Q4: Regarding the strong implications for histone degradation brought forward by the authors: It appears that experimental evidence should be provided by the authors.

***Response****:* We thank the reviewer for pointing out this issue. Although we do not provide our own experimental evidence, the importance of PA200-S20 proteasome for histone degradation has been confirmed in several earlier studies, which we cite in our manuscript. Qian et al. were among the first to present substantial evidence that proteasomes containing the activator PA200 catalyze the polyubiquitin-independent degradation of histones during DNA double-strand breaks and delay core histone disappearance in elongated spermatids. Another study demonstrated that recombinant PA200 uses its bromodomain-like region to bind acetylated H4 histones and promote their ATP-independent degradation [13]. Finally, a more recent study by Imke et al. confirmed that the PA200-bound proteasome indeed mediates the degradation of acetylated histones induced by DNA damage [14].

**Rev. 2:**

Q1: From the description and the work it is not fully clear if the identification of IP4 and IP6 is fully confident based on the density maps. Cleary it has not been experimentally addressed or confirmed, so would some caution in the conclusion be valid?

***Response****:* We thank the reviewer for this kind suggestion. When we obtained the density maps of PA200 and PA200-20S, we noticed that two well-defined densities are partly covering the central channel and interacting with the side-chains of several positively charged residues. The small molecule bound in the opening 2 has symmetrically arranged six side groups resembling phosphonates, so we assumed that the molecule is inositol phosphate. When making this assumption, we considered that previous studies have shown that Ins(1,4,5,6)P4 is a bona fide conserved regulator of Class I histone deacetylases (HDACs) that acts as an ‘intermolecular glue’ between HDAC3 and its co-repressor SMRT [10-12]. Since PA200 plays an essential role in the degradation of acetylated histones [13, 14], we modeled inositol phosphates into the density maps and refined them. The data indicated that the densities at the opening 1 are compatible with a molecule of 5,6[PP]2-InsP4, while the density at the opening 2 is more likely to represent InsP6.

As suggested by the reviewer, we carefully recalculated the densities of these two small molecules and are convinced that they represent two different molecules. To validate our structural data, standard InsP6 (phytic acid sodium salt hydrate, C_6_H_18_O_24_P_6_·xNa^+^·yH_2_O, MERCK, P8810) and purified PA200 (20 mM HEPES, pH 7.5 and 150 mM NaCl, 1mM DTT) were boiled for 5 minutes at 95°C and analyzed by HPLC-MS. The standard InsP6 produced a characteristic peak at 814.85 (M_InsP6_+7Na)^+^ (**please see the new Fig 6A**), and PA200 produced several characteristic peaks. In addition to the characteristic peak of 814.85 (M_InsP6_+7Na)^+^ in PA200, several other peaks representing different forms of sodium 5,6[PP]2-InsP4 with varying number of Na^+^ were observed, such as peak 1019.19 (M_5,6[PP]2-InsP4_+9Na)^+^, peak 1041.13 (M_5,6[PP]2-InsP4_+10Na)^+^ and peak 1063.24 (M_5,6[PP]2-InsP4_+11Na)^+^ (**please see the new Fig 6B**).

Considering all the evidence, we conclude that the two small molecules blocking/binding opening 1 and opening 2 are 5,6[PP]2-InsP4 and InsP6, respectively. The paragraph describing these results were added under "**Two openings on PA200 bind inositol phosphate cofactors**" subsection of the results.

Q2: Line 198. PA200 can interact with three complexes: Does PA200 not associate with immature CP complexes? The yeast orthologue does.

***Response****:* Indeed, previous studies have shown that Blm10 is found in association with CP precursor complexes and specifically promotes proteasome maturation [15, 16]. Similarly, PA200 does not only form stable complex with mature and active 20S CP but also with precursors or nascent species [17].

Q3: Line 297 “binds acetylated histones with “as key residues.” & line 305. Explain in more detail, authors do not show this, they propose this? Or references? Line 313 PA200 blm10 unlikely to bind acetylated histone based on this structure? How is the binding site of the acetylated histone related to the two channels described? Does the structure provide any clues towards the weird proposed ability of PA200-20S to be able to degrade histones?

***Response****:* We thank the reviewer for pointing out these issues. The key residues that the BRDLs of PA200 and Blm10 use to bind acetylated histones have been previously reported [13]. Also, we have followed the suggestion to add references at line 323.

To resolve the ambiguity caused by unclear description that led to the conclusion in the line 313 of the original manuscript, we rewrote the description and the conclusion to read as follows (lines 330–343 of the revised manuscript): " Both primary sequence and structural alignment of PA200 and Blm10 show that their BRDLs — aa 1650–1731 in PA200 (**Fig 7C, magenta**) and aa 1650-1732 in Blm10 (**S5C Fig, red**) — could not be aligned with each other. Instead, the BRDL of PA200 corresponds to the aa 1961–2042 region of Blm10 (**S5C Fig, cyan**), whereas the aa 1356–1432 region (**Fig 7C, yellow**) of PA200 corresponds to the BRDL of Blm10. The structural alignment of the PA200 BRDL with the cyan region of Blm10 gave the RMSD of 1.32 Å (70 Cα atoms), whereas the structural alignment of the Blm10 BRDL with the yellow region of PA200 gave RMSD of 2.11 Å (78 Cα atoms). In spite of the seeming correspondence of primary sequence and structure alignment, the yellow region of PA200 and cyan region of Blm10 do not share any sequence homology with BRD domains and do not possess a typical motif for reading acetyl-lysines, so we conclude that they are not true counterparts to the BRDLs of PA200 and Blm10, and are thus unlikely to mediate recognition of acetylated histones. "

Regarding the relationship between acetylated histone binding site and the two openings, PA200 spirals through a 1.5 turn left-handed solenoid resembling a snail shell and caps 20S. PA200 interacts with all seven α subunits of the 20S, but its spiral architecture causes the contact between the two molecules to be asymmetrical, with with a lateral opening (red dashed line box) situated over the subunits α1, α6 and α7. BRDL domain (red), which sits at the platform formed by α1, α6 and α7 subunits, forms a groove at the C-terminal end of PA200 together with neighboring region. The key residues (blue) reported to read the acetylated lysines at the N-terminal tail of H4 histone are located at the end of the groove and they are physically close to the opening 1 (cyan). Considering the architectures of PA200 and the H4 histone, we propose that the groove with BRDL domain facilitates the binding of H4 histone tails.

Knowing that it would provide the most precise picture of the relationship between the openings and acetylated histone binding and degradation, we also attempted to obtain the structures of PA200-acetylated H4 histone or BRDL-acetylated H4 histone complex. Unfortunately, we failed and were compelled to discuss the relationship on the basis of our structural data and relevant literature. The failure could possibly be explained by generally low affinities of Kac for BRDs, particularly in case of a single Kac site [18]. In addition, the BRDLs of Blm10 and PA200 are speculated to require additional posttranslational modifications to bind acetyl-histones [13].

Q4: The authors describe a PA200 induced increase in Vmax. Do they believe this is from gate opening? Also, the authors ignore the Km change they observe. The change in Km would suggest a change in the substrate binding environment, which would be consistent with the change observed in the recent Molecular cell paper?

***Response****:* The α-ring gates of 20S proteasomes were proposed to be dynamic and to allow substrates access into the catalytic chamber even without association with RP or other proteasomal activators [19]. This is consistent with the previous observation that free 20S proteasomes exhibit physiologically relevant catalytic activity both in vivo and in vitro [20, 21] and our data (**Fig 3G**). Our results have also shown that the formation of PA200-20S complex can indeed stimulate the chymotryptic-like activity.

Earlier studies showed that the binding of endogenous PA200 from bovine testes with purified human 20S proteasomes could activate all three catalytic subunits and modulate the three peptidase activities of the 20S proteasome, especially the caspase-like activity [21]. They attributed this modulation to the opening of a proteasome axial gate which facilitates access of small peptides to the proteasome inner chamber. These results are consistent with the *Pf*PA28 which induced the improvement of all three catalytic activities of *Pf*20S [8]. However, the detailed molecular basis of allosteric modulation of the proteasome active sites has not yet been determined for any proteasome [7].

Ana et al. recently proposed that PA200 activated the trypsin-like activity and slightly inhibited caspase-like and chymotryptic-like activities of the proteasome by allosterically modulating the 20S proteasome active sites. They attributed the changes in the three activities to slight differences in the lining of the three substrate-binding pockets between the uncapped 20S proteasome and 20S-PA200 complex. However, the modulation described in their paper is inconsistent with the details previously reported by Ustrell et al [21].

To verify whether the Km change is related in substrate binding environment, we compared the three active sites in our PA200-20S structure (6KWY) with those in the structures of PA200_2_-20S (6REY) and 20S (6RGQ) detailed in the Molecular Cell paper and the structure of 20S (5LE5) with a 1.9Å resolution. We could not clearly observe the subtle conformational changes of the three active sites neither when comparing differences between PA200-20S and 20S structures of their structures nor when comparing their structures with our structure. Moreover, we observed notable inconsistencies between the surface charge distribution of the β2 active site in all structures (PDB: 5LE5, 6RGQ, 6REY and 6KWY) when the images were generated by ourselves (please see the figures below, β2, dotted rectangle) and the corresponding figure (Figure 7) in the Molecular Cell article. Altogether, we were inclined to propose that PA200 binding causes the increase in three activities due to induced proteasome gate opening rather than by allosteric modulation of the three active sites.

Q5: The language and description of the paper is overall good, however, some care could be taken to polish it further. E.g. the improper use of significant, see e.g. line 43, line 64.; line 50 replace “especially” with “and particularly high” or something like that. Line 51 complexES. Line 225 has -> have.

***Response****:* We thank the reviewer for pointing this out. We carefully improved the language and revised any improper use of words. For example, the term "significant" was replaced with "important" at line 46 and "essential" at line 65, the word "especially" was replaced with "and particularly high" at line 52 etc.

Q6: Line 209: conformational change not clear from picture to me.

***Response****:* Though PA200 can interact with all seven α-subunits of the 20S, the 1.5 turn left-handed spiral structure of PA200 causes the contacts between the two molecules to be asymmetrical, with a lateral opening largely situated over the subunits α1, α6 and α7 (**Fig 2F**). A 23Å resolution EM study gave the notion that only first six α subunits (α1–α6) interact with PA200 and the subunit α7 does not. However, with the assistance of our high-resolution structures, we found that the α7 subunit can interact with PA200 through its N-terminal loop after the conformational change induced by binding of the with PA200, and that the subunits α1, α6 and α7 have a smaller contact area than the other 4 subunits (**Fig 2F, 4A and 4F**).

**Rev. 3:**

Q1: The authors state in the introduction that this manuscript follows the recent publication of another high resolution cryo-EM study of human PA200-20S complexes. Most of the features described in their manuscript have already been presented there. Accordingly, they should revise the presentation of their results in the light of the published data. Starting from the current title, with the reference to the “regulation of proteasome gate opening and two novel PA200 apertures”, the authors refer to features that are presented as new, but which unfortunately have already been described. This continues throughout the manuscript. The authors must clarify what is really the new information obtained from their analysis and acknowledge what is already known.

***Response****:* We thank the reviewer for this kind suggestion. Although the results presented in study by Ana et al. and our research are partially overlapping, the two studies were finished roughly at the same time and are in support of each other by obtaining overlapping results through distinct experimental approach. Moreover, the two studies describe these results from two different perspectives. To clarify the new information gained from our analysis and acknowledge what is already known, we changed the description of some sentences and cited the Mol Cell paper when we were analyzing and interpreting our data. Moreover, we compared our study with the one by Ana et al. in a newly added paragraphs of the Discussion (lines 444–453): "During our manuscript submission process, another structural study on the 20S and PA200-20S proteasome complex by Ana et al. was published in Molecular Cell. The study is highlighted by a successful assembly of the eukaryotic proteasome by recombinant expression and structures of uncapped 20S and double capped PA2002-20S complex. Moreover, the article discusses the allosteric modulation of 20S proteasome active sites by PA200. Similarly to our study, the authors also identify the two openings on PA200 and observe that they bind inositol phosphate cofactors. Although the scopes and results of the two studies partially overlap, the data is complementary and serves to support the findings of either study and is described and analyzed from two different perspectives to give a better all-around picture of the subject. "

We thank the reviewer for his remark and hope that the novelty in our study is now presented clearly to all readers.

Q2: The authors assembled their PA200-20S complexes by adding recombinant PA200 to an endogenous commercially available 20S. The electron microscope images in Figures 1A and 1B show significant heterogeneity of the samples obtained. Could this be improved by an additional size exclusion step? The authors should also clarify if they expressed PA200 in insect or bacterial cells, as both are referred to in the methods.

***Response:*** We apologize for the ambiguity in our description of the expression of human PA200. In order to provide the reader with a more accurate description of the experimental procedure and more details about it, we rewrote the relevant subsection of Methods (lines 844-861 of the revised manuscript).

Initially, we tried to obtain the [homogeneous](https://cn.bing.com/dict/search?q=homogeneous&FORM=BDVSP6&mkt=zh-cn) PA200-20S or PA200_2_-20S by incubating excess PA200 with 20S CP, but the homogeneity of the samples could not significantly be improved by only increasing the PA200 concentration. The increase of molar ratios between PA200 and 20S CP from 4.4:1 to 8.8:1 only led to increase in the proportion of PA200_2_-20S from 33.3% to 47.6%, and reduced the proportion of PA200-20S from 51.0% to 40.2%. Although we could obtain sufficient amounts of PA200 (~2 mg/liter of infected culture) by expressing it in the insect cells, commercially available 20S CP was the limiting factor (50 μg/[package](https://cn.bing.com/dict/search?q=package&FORM=BDVSP6&mkt=zh-cn), Boston Biochem, lot. #16518518) to get enough incubated sample for further gel filtration purification, which made it difficult to separate the PA200-20S and PA200_2_-20S complexes by gel filtration. However, thanks to the advances in cryo-electron microscopy, 3 μg of the heterogeneous sample were sufficient for us to carry out structural data collection and pick out the particles that interested us for structure determination.

Q3: Figures 1A and 1B also show that the PA200 and 20S binding efficiency is not very good. Could this be due to biochemical related issues, such as the use of non-fresh 20S? Additionally, since it is well known that particle picking software favors the recognition of globular particles, rather than more elongated ones like PA2002 20S, it is not clear if the different ratios of uncapped, single-capped and double-capped complexes obtained are physiologically significant.

***Response****:* We thank the reviewer for pointing this out. The previous studies have shown that uncapped, single-capped and double-capped proteasomes co-exist both *in vivo* and *in vitro* conditions. However, the physiological function of such complex heterogeneity is still poorly understood [13].

In our study, we were unable to obtain homogeneous samples of neither PA200-20S nor PA200_2_-20S at any tested molar ratio between PA200 and 20S, even when it was as high as 8.8:1. We believe that two possible reasons prevented us from obtaining homogeneous complex samples:

1. Saturation of 20S capping [can't](https://zhidao.baidu.com/question/490908447488130972.html) be achieved by merely increasing the PA200 concentration.

Under physiological conditions, proteasomes in eukaryotes, archaea, and some bacteria need to bind proteasomal activators at either one or both ends of the proteasome CP for full activity [22]. Hybrid proteasomes with different activators on either end of the CP cylinder have also been observed [23]. Moreover, more than 50% of the cellular 20S proteasomes exist in an activator-unbound form [24]. Altogether, these studies suggest that proteasomes indeed exist as a mixture of uncapped, single-capped and double-capped complexes under physiological conditions, albeit for unknown reasons. With regard to PA200, our findings are supported by the recently published article in which the complexes were prepared by co-expressing the two proteins in insect cells. In spite of different experimental procedure for protein expression and purification, their cryo-EM images also showed varying proportions of uncapped, single-capped and double-capped complex particles, with 28% of the particles in the final volume classified as single-capped complex [7].

2. The freshness of the protein sample.

As it is already known, the binding efficiency of freeze-thawed proteins is sometimes impacted at [varying](https://cn.bing.com/dict/search?q=varying&FORM=BDVSP6&mkt=zh-cn) [degree](https://cn.bing.com/dict/search?q=degree&FORM=BDVSP6&mkt=zh-cn)s.The 20S protein sample used to prepare the complex is an endogenously expressed commercial protein.

When we processed the data, the particles were auto picked based on the templates from initial 2D class revealing the average results of negative staining (which were used for titration of the 20S and PA200) using Gautomatch. Based on the 2D class average results of cryo-EM images, we were confident that we obtained sufficient number of diversified views of the 20S and single-capped complex, and showing insignificantly preferred orientations. However, we could not obtain enough of double-capped complexes for 3D reconstruction due to the dynamic binding between PA200 and 20S, possibly due to binding dynamics between PA200 and 20S, and, to a less extent, the loss of double-capped complexes during the cryogenic sample preparation process (**please see the new** **flow chart, Fig S1**).

To conclude, we believe that binding dynamics plays a major role in the observed binding efficiency between PA200 and 20S, but the freshness of the 20S sample may also impact binding to a certain degree.

Q4: The authors “refined the resolution of PA200-20S complex to 2.72 A via block-based reconstruction by using the threshold value of 0.143 for the gold-standard Fourier shell correlation”. These procedures were developed to deal with Ewald sphere effects in the reconstruction of large particles, such as viruses, and it would be interesting for the reader if the authors would clarify why these procedures were selected. Did the authors use the Relion software for this? Following the Relion workflow, the authors also did post-processing using a mask corresponding to a “3-pixel extension and 3-pixel fall-off around the entire molecule”. Because this is a quite tight and sharp mask could the authors discuss how they avoided overfitting? Why did the maps obtained with Relion needed validation using cisTEM? Could other validation approaches be used?

***Response****:* We agree with the reviewer that the block-based reconstruction method was originally developed for correcting various defocus values of different parts of large particles such as viruses. However, we found that this method could also slightly improve densities of other molecules, such as the PA200-20S complex presented in our project. It is convenient to apply this method in RELION3.0 after correcting defocus values using customized scripts. For this project, we roughly classified the 20S and single-capped PA200-20S using 2D classification, but we could not distinguish the uncapped and single-capped complex from the top view (globular particles). We therefore, combined all top view particles with single-capped PA200-20S side view particles to reconstruct high-resolution structure, then classified the 20S top view particles using this method and combined with 20S side view particles to reconstruct the 20S density map. By doing so, we improved the quality of the single-capped PA200-20S density.

As for the mask in post-processing, we referred to the tutorial of RELION and set the extended and soft-edged pixels to 3. Since we created the mask from lowpass filtered map (15 Å) and used a small enough binarization threshold, this mask could be solvent enough for avoiding overfitting. To verify the reconstructed map, we first applied the standard processing procedure using RELION and tested the dataset using cisTEM, which reported a little higher resolution, but the map was slightly ambiguous for modeling in COOT, so the RELION map was eventually used for modeling and further analysing.

Q5: The authors mentioned they determined the structures of both PA200-20S and PA200 on its own. However, they do not present the structure of PA200 in detail, nor they discuss it including regarding any conformational changes that may occur upon its binding to 20S.

***Response****:* We thank the reviewer for pointing this out. The details of the free PA200 structure had been presented in the first paragraph of the "Overall structures of the human PA200 and PA200-20S complex" section. Additionally, we have followed the suggestion and added two panels (**please see the new Fig 2G and 2H**) and a paragraph to discuss the conformational changes between free PA200 and that from the PA200-20S complex.

The paragraph reads as follows (lines 213-217):

"Although the resolution of PA200 was much lower than PA200-20S, the overall comparison of free PA200 and that from the PA200-20S complex gave RMSD of 0.78 Å with 1719 aligned residues. Some regions (i.e. residues 562–575, 1809–1843) vanished in PA200 but were clear in the PA200-20S complex, suggesting that these flexible regions tend to be stabilized by intermolecular interactions (**Fig** **2G and 2H**)."

Q6: The authors monitored the interaction of PA200 with the 20S by measuring its chymotryptic-like activity. However, others reported that PA200 enhances the tryptic- or caspase-like activities instead. This needs to be discussed.

***Response****:* The α-ring gates of 20S proteasomes were proposed to be dynamic and allowed access of substrate to the catalytic chamber even without the attachment of the RP or other proteasomal activators [19]. This is consistent with the previous observation that free 20S proteasomes exhibit physiologically relevant catalytic activity both in vivo and in vitro [20, 21] and to our data (**Fig 3G**). Our result also has shown that the formation of PA200-20S complex could stimulate the chymotryptic-like activity.

Earlier studies showed the binding of endogenous PA200 from bovine testes with purified human 20S proteasomes can activate all the three catalytic subunits and modulate the three peptidase activities of the 20S proteasome, especially the caspase-like activity [21]. They attributed this modulation to the opening of a proteasome axial gate which facilitates access of small peptides to the proteasome inner chamber. These results are consistent with the *Pf*PA28 which induced the improvement of all three catalytic activity of *Pf* 20S [8].

However, Ana et al. proposed that PA200 allosterically modulated the 20S proteasome active sites, significantly activated the proteasome trypsin-like activity, and slightly inhibited its caspase and chymotryptic-like activities. They attributed the differences between the three activities to a slight variation in the lining of the three proteasome substrate-binding pockets in their 20S proteasome and 20S-PA200 complex [7].

We compared the three active sites in our PA200-20S structure (6KWY) to that in the structures of PA200_2_-20S (6REY) and 20S (6RGQ) in the Molecular cell paper and the structure of 20S (5LE5) with a 1.9Å resolution. However, the slight conformational changes between the active sites in their PA200_2_-20S and 20S structures are not readily observable. Similarly, the conformational changes of the active sites in our structure were also not conspicuous. Moreover, we observed notable inconsistencies between the surface charge distribution of the β2 active site in all structures (PDB: 5LE5, 6RGQ, 6REY and 6KWY) when the images were generated by ourselves (please see the figures below, β2, dotted rectangle) and the corresponding figure (Figure 7) in the Molecular Cell article. The catalytic activity of 20S induced by PA200 thus requires further investigation.

Q7: The authors describe a PA200 BRD domain that “can't be classified into either of the eight typical human BRD families”. This is very much based on the sequence analysis of PA200, but what is the evidence that this novel domain has indeed a BRD function?

***Response****:* We appreciate the reviewer’s comment on this interesting and vital point. In 2012, Panagis et al. defined 61 BRDs in the human genome located in 46 diverse proteins and clustered into eight distinct families [18]. Qian et al. described the region aa1648-1732 of yeast Blm10 and aa1650-1738 of human PA200 as BRDL (BRD-like) region. Their results have shown that BRDL region of Blm10 could specifically pull down the histones from rabbit thymus which were further acetylated by Gcn5 *in vitro*. The mutations within this region (Y1663H/N1664D) abolished histone binding. Similarly, the BRDL region aa1650-1738 of human PA200, but not its corresponding mutant form (N1716T/F1717S) or a non-BRDL region of PA200 was found to bind acetyl-histones directly. The overall [conclusion](https://cn.bing.com/dict/search?q=conclusion&FORM=BDVSP6&mkt=zh-cn) was that BRD-like regions of Blm10 and PA200 could bind acetyl-histones *in vitro*, but additional posttranslational modifications of histones might be required to assist the binding [13].

Our structure has shown that although the BRDL region of PA200 does not share any significant sequence homology with known typical human BRDs, it folds into the same geometry. Thus, we proposed that the BRDL region of PA200 is not a canonical BRD. Future structural studies of the PA200 (or PA200-20S)-histone (or H4 N-terminal tail) complex will help us to understand the mechanism by which PA200 uses its BRDL domain to bind and read the key acetylated residues and subsequently mediate the degradation of the substrate in association with the 20S.

Q8: The representation of the experimental structures needs significant improvement, to allow the reader to appreciate their quality and the models built. As an example, in Figure 5D a slice of the full map densities should be shown, rather than just showing the densities of interest, so that the quality and strength of the densities for the cofactors could be fully appreciated.

***Response****:* We appreciate the reviewer’s suggestion and have added a slice of full map densities to the overall structures of PA200 (**Fig S7A**) and PA200-20S (**Fig S7B**) as well as to significant protein regions discussed in our study, such as the two openings on PA200 and inositol phosphate cofactors (**Fig S7C and S7D**), the loop 3 (**Fig S7E**) and BRDL domain of PA200 (**Fig S7F**). We replaced the previous panels B and D of Fig 3 with new panels that show close-up views of the top and bottom α-rings of single-capped 20S as cryo-EM map (gray mesh) with a fitted atomic model (cartoon representation) (**please see the new Fig 3**). We also added the **Fig S3** to our supplementary data for comparison of 20S core particle gate in PA200-bound and uncapped state. Panels A and B of the figure show close-up views of cryo-EM map (gray mesh) with a fitted atomic model (cartoon representation) of the top and bottom α-rings of uncapped 20S (**please see the new Fig S3**).

***Reference:***

1.	Toste Rêgo A, da Fonseca PCA. Characterization of Fully Recombinant Human 20S and 20S-PA200 Proteasome Complexes. Molecular Cell. 2019. doi: 10.1016/j.molcel.2019.07.014.

2.	Schmidli C, Albiez S, Rima L, Righetto R, Mohammed I, Oliva P, et al. Microfluidic protein isolation and sample preparation for high-resolution cryo-EM. Proc Natl Acad Sci U S A. 2019;116(30):15007-12. doi: 10.1073/pnas.1907214116. PubMed PMID: 31292253; PubMed Central PMCID: PMCPMC6660723.

3.	Groll M, Ditzel L, Lowe J, Stock D, Bochtler M, Bartunik HD, et al. Structure of 20S proteasome from yeast at 2.4 A resolution. Nature. 1997;386(6624):463-71. doi: 10.1038/386463a0. PubMed PMID: 9087403.

4.	Unno M, Mizushima T, Morimoto Y, Tomisugi Y, Tanaka K, Yasuoka N, et al. The structure of the mammalian 20S proteasome at 2.75 A resolution. Structure. 2002;10(5):609-18. doi: 10.1016/s0969-2126(02)00748-7. PubMed PMID: 12015144.

5.	Huber EM, Basler M, Schwab R, Heinemeyer W, Kirk CJ, Groettrup M, et al. Immuno- and constitutive proteasome crystal structures reveal differences in substrate and inhibitor specificity. Cell. 2012;148(4):727-38. doi: 10.1016/j.cell.2011.12.030. PubMed PMID: 22341445.

6.	Harshbarger W, Miller C, Diedrich C, Sacchettini J. Crystal structure of the human 20S proteasome in complex with carfilzomib. Structure. 2015;23(2):418-24. doi: 10.1016/j.str.2014.11.017. PubMed PMID: 25599644.

7.	Toste Rego A, da Fonseca PCA. Characterization of Fully Recombinant Human 20S and 20S-PA200 Proteasome Complexes. Mol Cell. 2019;76(1):138-47 e5. doi: 10.1016/j.molcel.2019.07.014. PubMed PMID: 31473102.

8.	Xie SC, Metcalfe RD, Hanssen E, Yang T, Gillett DL, Leis AP, et al. The structure of the PA28-20S proteasome complex from Plasmodium falciparum and implications for proteostasis. Nat Microbiol. 2019;4(11):1990-2000. doi: 10.1038/s41564-019-0524-4. PubMed PMID: 31384003.

9.	Rabl J, Smith DM, Yu Y, Chang SC, Goldberg AL, Cheng Y. Mechanism of gate opening in the 20S proteasome by the proteasomal ATPases. Mol Cell. 2008;30(3):360-8. doi: 10.1016/j.molcel.2008.03.004. PubMed PMID: 18471981; PubMed Central PMCID: PMCPMC4141531.

10.	Watson PJ, Fairall L, Santos GM, Schwabe JW. Structure of HDAC3 bound to co-repressor and inositol tetraphosphate. Nature. 2012;481(7381):335-40. doi: 10.1038/nature10728. PubMed PMID: 22230954; PubMed Central PMCID: PMCPMC3272448.

11.	Watson PJ, Millard CJ, Riley AM, Robertson NS, Wright LC, Godage HY, et al. Insights into the activation mechanism of class I HDAC complexes by inositol phosphates. Nat Commun. 2016;7:11262. doi: 10.1038/ncomms11262. PubMed PMID: 27109927; PubMed Central PMCID: PMCPMC4848466.

12.	Millard CJ, Watson PJ, Celardo I, Gordiyenko Y, Cowley SM, Robinson CV, et al. Class I HDACs share a common mechanism of regulation by inositol phosphates. Mol Cell. 2013;51(1):57-67. doi: 10.1016/j.molcel.2013.05.020. PubMed PMID: 23791785; PubMed Central PMCID: PMCPMC3710971.

13.	Qian MX, Pang Y, Liu CH, Haratake K, Du BY, Ji DY, et al. Acetylation-mediated proteasomal degradation of core histones during DNA repair and spermatogenesis. Cell. 2013;153(5):1012-24. doi: 10.1016/j.cell.2013.04.032. PubMed PMID: 23706739; PubMed Central PMCID: PMCPMC3983474.

14.	Mandemaker IK, Geijer ME, Kik I, Bezstarosti K, Rijkers E, Raams A, et al. DNA damage-induced replication stress results in PA200-proteasome-mediated degradation of acetylated histones. EMBO Rep. 2018;19(10). doi: 10.15252/embr.201745566. PubMed PMID: 30104204; PubMed Central PMCID: PMCPMC6172457.

15.	Fehlker M, Wendler P, Lehmann A, Enenkel C. Blm3 is part of nascent proteasomes and is involved in a late stage of nuclear proteasome assembly. EMBO Rep. 2003;4(10):959-63. doi: 10.1038/sj.embor.embor938. PubMed PMID: 12973301; PubMed Central PMCID: PMCPMC1326396.

16.	Gu ZC, Enenkel C. Proteasome assembly. Cell Mol Life Sci. 2014;71(24):4729-45. doi: 10.1007/s00018-014-1699-8. PubMed PMID: 25107634.

17.	Savulescu AF, Glickman MH. Proteasome activator 200: the heat is on. Mol Cell Proteomics. 2011;10(5):R110 006890. doi: 10.1074/mcp.R110.006890. PubMed PMID: 21389348; PubMed Central PMCID: PMCPMC3098604.

18.	Filippakopoulos P, Picaud S, Mangos M, Keates T, Lambert JP, Barsyte-Lovejoy D, et al. Histone recognition and large-scale structural analysis of the human bromodomain family. Cell. 2012;149(1):214-31. doi: 10.1016/j.cell.2012.02.013. PubMed PMID: 22464331; PubMed Central PMCID: PMCPMC3326523.

19.	Osmulski PA, Hochstrasser M, Gaczynska M. A tetrahedral transition state at the active sites of the 20S proteasome is coupled to opening of the alpha-ring channel. Structure. 2009;17(8):1137-47. doi: 10.1016/j.str.2009.06.011. PubMed PMID: 19679091; PubMed Central PMCID: PMCPMC2746003.

20.	Liu CW, Corboy MJ, DeMartino GN, Thomas PJ. Endoproteolytic activity of the proteasome. Science. 2003;299(5605):408-11. doi: 10.1126/science.1079293. PubMed PMID: 12481023; PubMed Central PMCID: PMCPMC3516294.

21.	Ustrell V, Hoffman L, Pratt G, Rechsteiner M. PA200, a nuclear proteasome activator involved in DNA repair. EMBO J. 2002;21(13):3516-25. doi: 10.1093/emboj/cdf333. PubMed PMID: 12093752; PubMed Central PMCID: PMCPMC126083.

22.	Tomko RJ, Jr., Hochstrasser M. Molecular architecture and assembly of the eukaryotic proteasome. Annu Rev Biochem. 2013;82:415-45. doi: 10.1146/annurev-biochem-060410-150257. PubMed PMID: 23495936; PubMed Central PMCID: PMCPMC3827779.

23.	Kunjappu MJ, Hochstrasser M. Assembly of the 20S proteasome. Biochim Biophys Acta. 2014;1843(1):2-12. doi: 10.1016/j.bbamcr.2013.03.008. PubMed PMID: 23507199; PubMed Central PMCID: PMCPMC3752329.

24.	Ben-Nissan G, Sharon M. Regulating the 20S proteasome ubiquitin-independent degradation pathway. Biomolecules. 2014;4(3):862-84. doi: 10.3390/biom4030862. PubMed PMID: 25250704; PubMed Central PMCID: PMCPMC4192676.
