# Supplementary material for: Cryo-EM structures of the human PA200 and PA200-20S complex reveal regulation of proteasome gate opening and two PA200 apertures
Source: PLoS Biol. 2020 Mar 5;18(3):e3000654. doi: 10.1371/journal.pbio.3000654 (PMC7077846; doi:10.1371/journal.pbio.3000654)
Supplement: Attachment — Submitted filename: Response to Reviewers.docx [file pbio.3000654.s013.docx]

1. Please note that your submission cannot contain any references to generic "supporting information" files and instead must refer to specific files using our naming convention (S1 Fig, S1 Table, S1 Text, S1 Data, etc.)

***Response:*** We removed the sentences: “All raw data and data processing of numerical values can be found in EXCEL files” (lines 700-701) and “All of the data can be found in the file of Supporting Information” (lines 701-702) in the manuscript. We also named all the supporting information files following the naming convention (S1 Fig, S1 Table, S1 Text, S1 Data, etc.) in the submission system.

2. Please provide data underlying Fig 3G, please mention where the data can be found in the legend for Fig 3.

***Response:*** We added the sentences: “Data underlying these plots for top panel can be found in [S1 Data](https://journals.plos.org/plosbiology/article?id=10.1371/journal.pbio.3000578#pbio.3000578.s001).” (lines 294-295) and “The underlying numerical data and statistical analysis for middle and bottom panels can be found in S2 and S3 Data.” (lines 297-298) to indicate where the data can be found in the legend for Fig 3G.

3. Please ensure that all main figure files follow PLOS naming conventions in the File Inventory. The file name should be in the format “Fig1.tif” and the file description should be “Fig 1”.

***Response:*** We confirmed that all main figure files follow PLOS naming conventions in the File Inventory of submission system which were named as “Fig1.tif” and described as “Fig 1”.
